# 5-*tert*-Butyl 3-ethyl 1-isopropyl-4,5,6,7-tetra­hydro-1*H*-pyrazolo­[4,3-*c*]pyridine-3,5-dicarboxyl­ate

**DOI:** 10.1107/S1600536811046332

**Published:** 2011-11-09

**Authors:** Huan-Mei Guo

**Affiliations:** a Microscale Science Institute, Weifang University, Weifang 261061, People’s Republic of China

## Abstract

In the title compound, C_17_H_27_N_3_O_4_, the six-membered ring adopts a half-chair conformation with the N atom and the adjacent methyl­ene C atom displaced by −0.391 (2) and 0.358 (2) Å, respectively, from the plane of the other four atoms. In the crystal, mol­ecules are linked by weak C—H⋯O inter­actions.

## Related literature

For a related structure and background references to heterocycles as pharmaceuticals, see: Guo (2011 ▶).
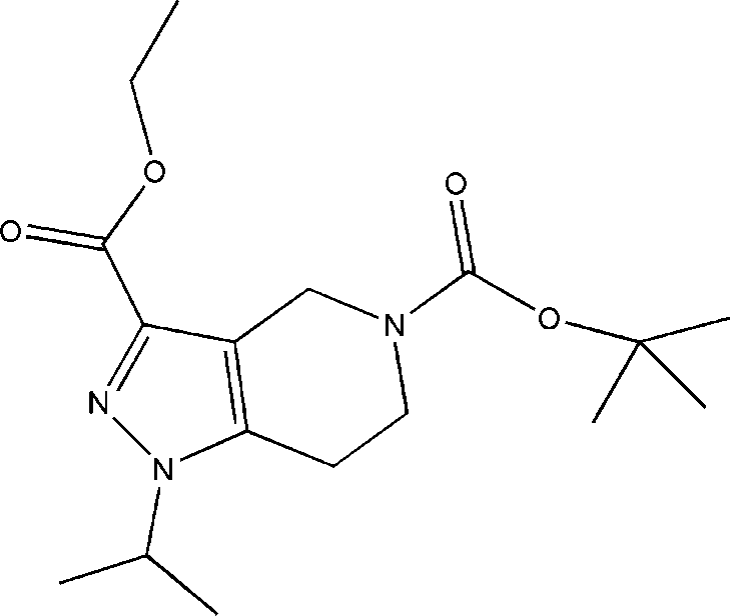


## Experimental

### 

#### Crystal data

C_17_H_27_N_3_O_4_
*M* *_r_* = 337.42
Monoclinic, 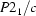

*a* = 13.017 (3) Å
*b* = 12.771 (3) Å
*c* = 11.952 (3) Å
β = 115.760 (3)°
*V* = 1789.4 (7) Å^3^
*Z* = 4
Mo *K*α radiation
μ = 0.09 mm^−1^
*T* = 173 K
0.21 × 0.17 × 0.06 mm

#### Data collection

Rigaku MM007-HF CCD (Saturn 724+) diffractometer
Absorption correction: multi-scan (*CrystalClear*; Rigaku, 2007 ▶) *T*_min_ = 0.981, *T*_max_ = 0.995
7844 measured reflections
4013 independent reflections
3330 reflections with *I* > 2σ(*I*)
*R*_int_ = 0.039

#### Refinement

*R*[*F* ^2^ > 2σ(*F* ^2^)] = 0.060
*wR*(*F* ^2^) = 0.128
*S* = 1.09
4013 reflections
223 parameters
H-atom parameters constrained
Δρ_max_ = 0.27 e Å^−3^
Δρ_min_ = −0.22 e Å^−3^

### 

Data collection: *CrystalClear* (Rigaku, 2007 ▶); cell refinement: *CrystalClear*; data reduction: *CrystalClear*; program(s) used to solve structure: *SHELXS97* (Sheldrick, 2008 ▶); program(s) used to refine structure: *SHELXL97* (Sheldrick, 2008 ▶); molecular graphics: *SHELXTL* (Sheldrick, 2008 ▶); software used to prepare material for publication: *SHELXTL*.

## Supplementary Material

Crystal structure: contains datablock(s) global, I. DOI: 10.1107/S1600536811046332/hb6476sup1.cif

Structure factors: contains datablock(s) I. DOI: 10.1107/S1600536811046332/hb6476Isup2.hkl

Supplementary material file. DOI: 10.1107/S1600536811046332/hb6476Isup3.cml

Additional supplementary materials:  crystallographic information; 3D view; checkCIF report

## Figures and Tables

**Table 1:** Hydrogen-bond geometry (Å, °)

*D*—H⋯*A*	*D*—H	H⋯*A*	*D*⋯*A*	*D*—H⋯*A*
C5—H5*B*⋯O1^i^	0.99	2.51	3.303 (3)	137
C11—H11*A*⋯O1^ii^	0.98	2.57	3.320 (3)	133

Symmetry codes: (i) 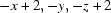; (ii) 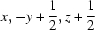.

**Fig. 1.**
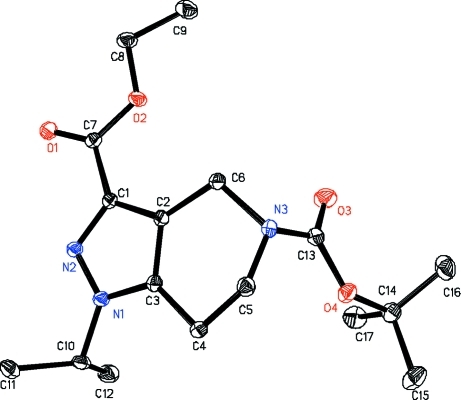
The molecular structure of the title compound with displacement ellipsoids drawn at the 30% probability level.

## supplementary crystallographic information

### Comment

As part of our ongoing studies of heterocycles (Guo, 2011), herein we report the structure of the title compound.

In the molecule (Fig. 1), all bond lengths and angles are within normal ranges.The atoms N1, N2, C1, C2, C3, C4 and C6 lie in a plan(p1), with a maximum deviation of 0.0100 (15)Å; atoms N3, C13, O3, O4, and C14 lie in a plan(p2) too, the maximum deviation is 0.0552 (12)Å. The dihedral angle between p1 and p2 is 75.44 (5)°.

### Experimental

The title compound was synthesized with 5-*tert*-butyl 3-ethyl 6,7-dihydro-1*H*- pyrazolo[4,3-*c*]pyridine-3,5(4*H*)- dicarboxylate (1 eq), NaH (2.5 eq) and 2-bromopropane (1.2 eq) in THF at 273 K to room temperature. Single crystals of the compound suitable for X-ray measurements were obtained by recrystallization from ethanol at room temperature.

### Refinement

All H atoms were fixed geometrically and allowed to ride on their attached atoms, with C—H distances in the range 0.98–1.00 Å, and with *U*_iso_(H) = 1.2*U*_eq_(C) or *U*_iso_(H) = 1.5*U*_eq_(C_methyl_).

### Crystal data

**Table:**

C_17_H_27_N_3_O_4_	*F*(000) = 728
*M**_r_* = 337.42	*D*_x_ = 1.252 Mg m^−^^3^
Monoclinic, *P*2_1_/*c*	Mo *K*α radiation, λ = 0.71073 Å
Hall symbol: -P 2ybc	Cell parameters from 5928 reflections
*a* = 13.017 (3) Å	θ = 1.6–27.5°
*b* = 12.771 (3) Å	µ = 0.09 mm^−^^1^
*c* = 11.952 (3) Å	*T* = 173 K
β = 115.760 (3)°	Block, colorless
*V* = 1789.4 (7) Å^3^	0.21 × 0.17 × 0.06 mm
*Z* = 4	

### Data collection

**Table:**

Rigaku MM007-HF CCD (Saturn 724+) diffractometer	4013 independent reflections
Radiation source: rotating anode	3330 reflections with *I* > 2σ(*I*)
Confocal	*R*_int_ = 0.039
ω scans at fixed χ = 45°	θ_max_ = 27.4°, θ_min_ = 1.7°
Absorption correction: multi-scan (*CrystalClear*; Rigaku, 2007)	*h* = −16→14
*T*_min_ = 0.981, *T*_max_ = 0.995	*k* = −16→15
7844 measured reflections	*l* = −8→15

### Refinement

**Table:**

Refinement on *F*^2^	Primary atom site location: structure-invariant direct methods
Least-squares matrix: full	Secondary atom site location: difference Fourier map
*R*[*F*^2^ > 2σ(*F*^2^)] = 0.060	Hydrogen site location: inferred from neighbouring sites
*wR*(*F*^2^) = 0.128	H-atom parameters constrained
*S* = 1.09	*w* = 1/[σ^2^(*F*_o_^2^) + (0.0346*P*)^2^ + 1.0152*P*] where *P* = (*F*_o_^2^ + 2*F*_c_^2^)/3
4013 reflections	(Δ/σ)_max_ = 0.001
223 parameters	Δρ_max_ = 0.27 e Å^−^^3^
0 restraints	Δρ_min_ = −0.22 e Å^−^^3^

### Special details

**Table:**

Geometry. All e.s.d.'s (except the e.s.d. in the dihedral angle between two l.s. planes) are estimated using the full covariance matrix. The cell e.s.d.'s are taken into account individually in the estimation of e.s.d.'s in distances, angles and torsion angles; correlations between e.s.d.'s in cell parameters are only used when they are defined by crystal symmetry. An approximate (isotropic) treatment of cell e.s.d.'s is used for estimating e.s.d.'s involving l.s. planes.
Refinement. Refinement of *F*^2^ against ALL reflections. The weighted *R*-factor *wR* and goodness of fit *S* are based on *F*^2^, conventional *R*-factors *R* are based on *F*, with *F* set to zero for negative *F*^2^. The threshold expression of *F*^2^ > σ(*F*^2^) is used only for calculating *R*-factors(gt) *etc*. and is not relevant to the choice of reflections for refinement. *R*-factors based on *F*^2^ are statistically about twice as large as those based on *F*, and *R*- factors based on ALL data will be even larger.

### Fractional atomic coordinates and isotropic or equivalent isotropic displacement parameters (Å^2^)

**Table:**

	*x*	*y*	*z*	*U*_iso_*/*U*_eq_	
O1	0.89479 (12)	0.21624 (10)	0.88381 (12)	0.0305 (3)	
O2	0.83497 (11)	0.09119 (10)	0.73679 (11)	0.0270 (3)	
O3	0.59568 (12)	−0.19414 (12)	0.66795 (13)	0.0379 (4)	
O4	0.62019 (11)	−0.32250 (10)	0.80972 (12)	0.0272 (3)	
N1	0.86219 (13)	−0.02179 (11)	1.09856 (13)	0.0214 (3)	
N2	0.87328 (12)	0.06944 (11)	1.04856 (13)	0.0208 (3)	
N3	0.77214 (13)	−0.23478 (12)	0.81927 (14)	0.0231 (3)	
C1	0.86041 (14)	0.04334 (13)	0.93459 (16)	0.0191 (4)	
C2	0.83954 (14)	−0.06483 (14)	0.91134 (16)	0.0194 (4)	
C3	0.84147 (14)	−0.10378 (14)	1.01911 (16)	0.0202 (4)	
C4	0.82646 (16)	−0.21603 (14)	1.04464 (17)	0.0236 (4)	
H4A	0.7507	−0.2267	1.0436	0.028*	
H4B	0.8859	−0.2369	1.1272	0.028*	
C5	0.83677 (16)	−0.28141 (14)	0.94246 (17)	0.0241 (4)	
H5B	0.9181	−0.2869	0.9595	0.029*	
H5A	0.8080	−0.3530	0.9433	0.029*	
C6	0.82002 (16)	−0.13484 (14)	0.80231 (17)	0.0245 (4)	
H6A	0.7665	−0.1010	0.7241	0.029*	
H6B	0.8930	−0.1474	0.7974	0.029*	
C7	0.86696 (14)	0.12674 (14)	0.85268 (16)	0.0206 (4)	
C8	0.83558 (18)	0.16686 (16)	0.64603 (17)	0.0307 (4)	
H8B	0.9146	0.1795	0.6575	0.037*	
H8A	0.8028	0.2342	0.6559	0.037*	
C9	0.7652 (2)	0.12184 (19)	0.51993 (18)	0.0409 (5)	
H9B	0.7695	0.1678	0.4565	0.061*	
H9A	0.6856	0.1159	0.5067	0.061*	
H9C	0.7943	0.0523	0.5141	0.061*	
C10	0.85336 (16)	−0.02244 (14)	1.21705 (16)	0.0241 (4)	
H10	0.8639	−0.0962	1.2482	0.029*	
C11	0.94661 (17)	0.04410 (16)	1.31347 (17)	0.0298 (4)	
H11B	1.0211	0.0209	1.3211	0.045*	
H11C	0.9441	0.0366	1.3939	0.045*	
H11A	0.9348	0.1177	1.2877	0.045*	
C12	0.73472 (17)	0.01270 (18)	1.19462 (19)	0.0365 (5)	
H12B	0.7229	0.0853	1.1652	0.055*	
H12C	0.7267	0.0079	1.2723	0.055*	
H12A	0.6779	−0.0324	1.1318	0.055*	
C13	0.65564 (16)	−0.24563 (15)	0.75820 (17)	0.0246 (4)	
C14	0.49694 (16)	−0.34246 (16)	0.76793 (18)	0.0293 (4)	
C15	0.49744 (19)	−0.43108 (18)	0.8521 (2)	0.0433 (6)	
H15A	0.4187	−0.4506	0.8330	0.065*	
H15C	0.5368	−0.4085	0.9389	0.065*	
H15B	0.5371	−0.4916	0.8389	0.065*	
C16	0.44174 (18)	−0.3756 (2)	0.6327 (2)	0.0434 (6)	
H16B	0.3628	−0.3971	0.6094	0.065*	
H16C	0.4843	−0.4345	0.6207	0.065*	
H16A	0.4425	−0.3167	0.5805	0.065*	
C17	0.44277 (19)	−0.24554 (19)	0.7929 (2)	0.0410 (5)	
H17A	0.4487	−0.1872	0.7428	0.062*	
H17C	0.4823	−0.2273	0.8811	0.062*	
H17B	0.3622	−0.2595	0.7708	0.062*	

### Atomic displacement parameters (Å^2^)

**Table:**

	*U*^11^	*U*^22^	*U*^33^	*U*^12^	*U*^13^	*U*^23^
O1	0.0424 (8)	0.0206 (7)	0.0280 (7)	−0.0067 (6)	0.0148 (6)	−0.0004 (5)
O2	0.0420 (8)	0.0206 (7)	0.0205 (6)	−0.0026 (6)	0.0154 (6)	0.0015 (5)
O3	0.0346 (8)	0.0423 (9)	0.0303 (8)	−0.0006 (7)	0.0079 (7)	0.0120 (7)
O4	0.0233 (7)	0.0266 (7)	0.0301 (7)	−0.0030 (5)	0.0099 (6)	0.0028 (6)
N1	0.0283 (8)	0.0172 (7)	0.0201 (7)	−0.0012 (6)	0.0119 (7)	0.0001 (6)
N2	0.0246 (8)	0.0177 (7)	0.0211 (7)	−0.0008 (6)	0.0108 (6)	0.0007 (6)
N3	0.0275 (8)	0.0192 (7)	0.0226 (8)	−0.0042 (6)	0.0110 (7)	−0.0027 (6)
C1	0.0179 (8)	0.0192 (9)	0.0202 (8)	−0.0008 (7)	0.0085 (7)	0.0001 (7)
C2	0.0199 (8)	0.0194 (9)	0.0198 (8)	−0.0022 (7)	0.0094 (7)	−0.0015 (7)
C3	0.0215 (9)	0.0183 (9)	0.0209 (8)	−0.0013 (7)	0.0092 (7)	−0.0012 (7)
C4	0.0294 (10)	0.0199 (9)	0.0219 (9)	−0.0032 (8)	0.0114 (8)	0.0010 (7)
C5	0.0263 (9)	0.0193 (9)	0.0248 (9)	−0.0005 (7)	0.0093 (8)	−0.0005 (7)
C6	0.0340 (10)	0.0205 (9)	0.0233 (9)	−0.0065 (8)	0.0164 (8)	−0.0037 (7)
C7	0.0191 (8)	0.0204 (9)	0.0225 (9)	0.0005 (7)	0.0093 (7)	0.0022 (7)
C8	0.0410 (12)	0.0279 (10)	0.0263 (10)	−0.0007 (9)	0.0176 (9)	0.0068 (8)
C9	0.0554 (14)	0.0414 (13)	0.0237 (10)	−0.0036 (11)	0.0152 (10)	0.0064 (9)
C10	0.0354 (10)	0.0223 (9)	0.0177 (8)	0.0003 (8)	0.0143 (8)	0.0017 (7)
C11	0.0368 (11)	0.0302 (11)	0.0210 (9)	0.0016 (9)	0.0113 (9)	−0.0022 (8)
C12	0.0343 (11)	0.0490 (13)	0.0320 (11)	−0.0023 (10)	0.0198 (9)	0.0007 (10)
C13	0.0285 (10)	0.0229 (9)	0.0231 (9)	−0.0023 (8)	0.0118 (8)	−0.0022 (8)
C14	0.0223 (9)	0.0337 (11)	0.0321 (10)	−0.0019 (8)	0.0121 (8)	−0.0006 (9)
C15	0.0356 (12)	0.0398 (13)	0.0555 (15)	−0.0079 (10)	0.0207 (11)	0.0071 (11)
C16	0.0289 (11)	0.0602 (16)	0.0395 (12)	−0.0135 (11)	0.0133 (10)	−0.0149 (11)
C17	0.0385 (12)	0.0445 (13)	0.0453 (13)	0.0071 (10)	0.0231 (11)	−0.0003 (11)

### Geometric parameters (Å, °)

**Table:**

O1—C7	1.208 (2)	C8—H8B	0.9900
O2—C7	1.340 (2)	C8—H8A	0.9900
O2—C8	1.455 (2)	C9—H9B	0.9800
O3—C13	1.214 (2)	C9—H9A	0.9800
O4—C13	1.343 (2)	C9—H9C	0.9800
O4—C14	1.480 (2)	C10—C12	1.516 (3)
N1—N2	1.345 (2)	C10—C11	1.519 (3)
N1—C3	1.360 (2)	C10—H10	1.0000
N1—C10	1.470 (2)	C11—H11B	0.9800
N2—C1	1.340 (2)	C11—H11C	0.9800
N3—C13	1.375 (2)	C11—H11A	0.9800
N3—C5	1.468 (2)	C12—H12B	0.9800
N3—C6	1.472 (2)	C12—H12C	0.9800
C1—C2	1.412 (2)	C12—H12A	0.9800
C1—C7	1.474 (2)	C14—C15	1.512 (3)
C2—C3	1.371 (2)	C14—C16	1.516 (3)
C2—C6	1.508 (2)	C14—C17	1.517 (3)
C3—C4	1.496 (2)	C15—H15A	0.9800
C4—C5	1.533 (2)	C15—H15C	0.9800
C4—H4A	0.9900	C15—H15B	0.9800
C4—H4B	0.9900	C16—H16B	0.9800
C5—H5B	0.9900	C16—H16C	0.9800
C5—H5A	0.9900	C16—H16A	0.9800
C6—H6A	0.9900	C17—H17A	0.9800
C6—H6B	0.9900	C17—H17C	0.9800
C8—C9	1.497 (3)	C17—H17B	0.9800
			
C7—O2—C8	116.44 (14)	C8—C9—H9C	109.5
C13—O4—C14	120.61 (14)	H9B—C9—H9C	109.5
N2—N1—C3	112.56 (14)	H9A—C9—H9C	109.5
N2—N1—C10	120.08 (14)	N1—C10—C12	109.16 (15)
C3—N1—C10	126.43 (15)	N1—C10—C11	110.89 (15)
C1—N2—N1	104.24 (14)	C12—C10—C11	112.66 (17)
C13—N3—C5	120.72 (15)	N1—C10—H10	108.0
C13—N3—C6	116.71 (15)	C12—C10—H10	108.0
C5—N3—C6	113.42 (14)	C11—C10—H10	108.0
N2—C1—C2	111.82 (15)	C10—C11—H11B	109.5
N2—C1—C7	118.53 (15)	C10—C11—H11C	109.5
C2—C1—C7	129.64 (16)	H11B—C11—H11C	109.5
C3—C2—C1	104.29 (15)	C10—C11—H11A	109.5
C3—C2—C6	121.62 (16)	H11B—C11—H11A	109.5
C1—C2—C6	134.09 (16)	H11C—C11—H11A	109.5
N1—C3—C2	107.09 (15)	C10—C12—H12B	109.5
N1—C3—C4	126.83 (15)	C10—C12—H12C	109.5
C2—C3—C4	126.07 (16)	H12B—C12—H12C	109.5
C3—C4—C5	107.16 (14)	C10—C12—H12A	109.5
C3—C4—H4A	110.3	H12B—C12—H12A	109.5
C5—C4—H4A	110.3	H12C—C12—H12A	109.5
C3—C4—H4B	110.3	O3—C13—O4	125.96 (18)
C5—C4—H4B	110.3	O3—C13—N3	123.52 (17)
H4A—C4—H4B	108.5	O4—C13—N3	110.48 (15)
N3—C5—C4	111.58 (15)	O4—C14—C15	102.34 (15)
N3—C5—H5B	109.3	O4—C14—C16	110.48 (15)
C4—C5—H5B	109.3	C15—C14—C16	111.31 (19)
N3—C5—H5A	109.3	O4—C14—C17	108.74 (16)
C4—C5—H5A	109.3	C15—C14—C17	110.36 (18)
H5B—C5—H5A	108.0	C16—C14—C17	113.07 (18)
N3—C6—C2	108.50 (14)	C14—C15—H15A	109.5
N3—C6—H6A	110.0	C14—C15—H15C	109.5
C2—C6—H6A	110.0	H15A—C15—H15C	109.5
N3—C6—H6B	110.0	C14—C15—H15B	109.5
C2—C6—H6B	110.0	H15A—C15—H15B	109.5
H6A—C6—H6B	108.4	H15C—C15—H15B	109.5
O1—C7—O2	123.77 (16)	C14—C16—H16B	109.5
O1—C7—C1	125.12 (17)	C14—C16—H16C	109.5
O2—C7—C1	111.09 (15)	H16B—C16—H16C	109.5
O2—C8—C9	107.22 (16)	C14—C16—H16A	109.5
O2—C8—H8B	110.3	H16B—C16—H16A	109.5
C9—C8—H8B	110.3	H16C—C16—H16A	109.5
O2—C8—H8A	110.3	C14—C17—H17A	109.5
C9—C8—H8A	110.3	C14—C17—H17C	109.5
H8B—C8—H8A	108.5	H17A—C17—H17C	109.5
C8—C9—H9B	109.5	C14—C17—H17B	109.5
C8—C9—H9A	109.5	H17A—C17—H17B	109.5
H9B—C9—H9A	109.5	H17C—C17—H17B	109.5
			
C3—N1—N2—C1	−0.73 (19)	C3—C2—C6—N3	16.5 (2)
C10—N1—N2—C1	−170.41 (15)	C1—C2—C6—N3	−164.66 (18)
N1—N2—C1—C2	0.77 (19)	C8—O2—C7—O1	0.4 (3)
N1—N2—C1—C7	179.55 (14)	C8—O2—C7—C1	178.84 (15)
N2—C1—C2—C3	−0.5 (2)	N2—C1—C7—O1	8.1 (3)
C7—C1—C2—C3	−179.14 (17)	C2—C1—C7—O1	−173.39 (18)
N2—C1—C2—C6	−179.51 (18)	N2—C1—C7—O2	−170.33 (15)
C7—C1—C2—C6	1.9 (3)	C2—C1—C7—O2	8.2 (3)
N2—N1—C3—C2	0.4 (2)	C7—O2—C8—C9	−163.00 (16)
C10—N1—C3—C2	169.31 (16)	N2—N1—C10—C12	75.7 (2)
N2—N1—C3—C4	179.00 (16)	C3—N1—C10—C12	−92.4 (2)
C10—N1—C3—C4	−12.1 (3)	N2—N1—C10—C11	−49.0 (2)
C1—C2—C3—N1	0.07 (18)	C3—N1—C10—C11	142.90 (17)
C6—C2—C3—N1	179.20 (15)	C14—O4—C13—O3	−8.2 (3)
C1—C2—C3—C4	−178.52 (17)	C14—O4—C13—N3	174.12 (15)
C6—C2—C3—C4	0.6 (3)	C5—N3—C13—O3	166.25 (18)
N1—C3—C4—C5	−165.31 (16)	C6—N3—C13—O3	21.5 (3)
C2—C3—C4—C5	13.0 (2)	C5—N3—C13—O4	−16.0 (2)
C13—N3—C5—C4	−77.8 (2)	C6—N3—C13—O4	−160.74 (14)
C6—N3—C5—C4	67.98 (19)	C13—O4—C14—C15	−178.30 (17)
C3—C4—C5—N3	−44.5 (2)	C13—O4—C14—C16	63.1 (2)
C13—N3—C6—C2	97.36 (18)	C13—O4—C14—C17	−61.5 (2)
C5—N3—C6—C2	−49.90 (19)		

### Hydrogen-bond geometry (Å, °)

**Table:**

*D*—H···*A*	*D*—H	H···*A*	*D*···*A*	*D*—H···*A*
C5—H5B···O1^i^	0.99	2.51	3.303 (3)	137
C11—H11A···O1^ii^	0.98	2.57	3.320 (3)	133

Symmetry codes: (i) −*x*+2, −*y*, −*z*+2; (ii) *x*, −*y*+1/2, *z*+1/2.
